# Long-Term Respiratory Outcomes in Pediatric Patients Following COVID-19 Infection and Multisystem Inflammatory Syndrome in Children (MIS-C)

**DOI:** 10.3390/biomedicines14081819

**Published:** 2026-08-13

**Authors:** Tijana Grba, Mihail Basa, Jelena Visekruna, Stasa Krasic, Vladislav Vukomanovic, Aleksandar Sovtic

**Affiliations:** 1 Department of Pulmonology, Mother and Child Health Care Institute of Serbia, 11070 Belgrade, Serbia; tijana.grba@imd.org.rs (T.G.); mihail.basa@imd.org.rs (M.B.); jelena.visekruna@imd.org.rs (J.V.); 2 Department of Cardiology, Mother and Child Health Institute of Serbia, 11070 Belgrade, Serbia; stasa.krasic@imd.org.rs (S.K.); vladislav.vukomanovic@med.bg.ac.rs (V.V.); 3 Faculty of Medicine, University of Belgrade, 11000 Belgrade, Serbia

**Keywords:** COVID-19, multisystem inflammatory syndrome in children, cardiopulmonary exercise testing, respiratory function tests, exercise tolerance

## Abstract

**Background/Objectives:** Although SARS-CoV-2 infection is usually mild in children, some develop severe respiratory disease (RD) or multisystem inflammatory syndrome in children (MIS-C). We evaluated long-term respiratory symptoms, pulmonary function, and cardiopulmonary exercise performance in children recovering from SARS-CoV-2-related RD and MIS-C. **Methods:** This single-center prospective observational study included children hospitalized for SARS-CoV-2-related RD or MIS-C. Pulmonary function tests (PFTs) and cardiopulmonary exercise testing (CPET) were performed at a mean follow-up of 22.8 ± 9.8 months after hospitalization and compared between groups. **Results:** Forty-two children (25 RD, 17 MIS-C) were included. Persistent post-COVID symptoms were reported by 38.1% of participants and were more frequent in the RD group (56.0% vs. 11.8%; *p* < 0.01), with fatigue being the predominant symptom (*p* = 0.02). Pulmonary function and CPET parameters were comparable between groups, with no evidence of clinically significant ventilatory limitation or impaired aerobic capacity. VO_2_peak did not differ between groups. Multivariable regression identified the breathing reserve index and OUES normalized to body weight as strong positive predictors of aerobic capacity in both groups. Despite differences in follow-up duration, stratified analyses using a 24-month cut-off showed comparable physiological findings. **Conclusions:** Children demonstrated preserved pulmonary function and exercise capacity nearly two years after hospitalization for severe SARS-CoV-2-related RD or MIS-C. Despite these favorable objective findings, subjective symptoms persisted in a substantial subset of patients, particularly those recovering from RD, suggesting that symptom resolution may lag behind objective physiological recovery. OUES normalized to body weight and the breathing reserve index may serve as complementary markers of aerobic capacity.

## 1. Introduction

Since the start of the pandemic, SARS-CoV-2 has infected approximately 580 million people, resulting in 6.5 million deaths. Even though most children with typical SARS-CoV-2 infection showed mild symptoms, some experienced severe lung disease or postinfection multisystem inflammatory syndrome (MIS-C), a novel disease characterized by systemic hyperinflammation and shock, which may or may not affect the heart [1,2].

In adults, pulmonary function tests (PFTs) were still impaired for several months after the infection. In children this was not present in the majority of cases, with faster normalization of PFTs [3]. Although full clinical recovery was documented in most cases, some patients continue to have Long COVID or just malaise and fatigue during exercise. Long COVID is heterogeneous and may be attributed to different underlying pathophysiologic processes even in children, including organ damage, complications from a dysregulated inflammatory state, microvascular dysfunction, autoimmunity, and other potential causes [4]. However, the relationship between persistent symptoms and objective physiological abnormalities remains incompletely understood, particularly in children recovering from severe SARS-CoV-2-related illness. Decreased exercise tolerance, chronotropic incompetence, muscle deconditioning, and ventilatory limitation on cardiopulmonary exercise testing (CPET) were observed in adults with the cardiopulmonary phenotype of Long COVID disease and children with MIS-C [3,4]. Recently published research on respiratory outcomes in Dutch children showed lung function and CPET abnormalities that correlate with respiratory symptoms [5]. Nevertheless, long-term studies combining detailed pulmonary function testing, diffusion capacity, ventilation homogeneity, and cardiopulmonary exercise testing after severe pediatric SARS-CoV-2-related illness remain scarce.

Oxygen consumption (VO_2_) at peak exercise during CPET is a valuable marker of cardiopulmonary aerobic fitness. The slope of the semi-log plot of minute ventilation (V_E_) versus VO_2_—Oxygen Uptake Efficacy Slope (OUES) is an effort-independent marker for estimation of ventilation in regard to VO_2_ [6]. Even submaximal OUES can provide an objective measure of cardiorespiratory function reflecting the efficacy of ventilation. A steeper OUES indicates better efficacy of ventilation. Adjusted to body surface area (BSA) or body mass (BM), it is a sensitive marker of the efficacy of the oxygen extracted by the lungs and used on the periphery [6]. Another useful marker of ventilatory efficacy is the breathing reserve index (BRI), calculated from V_E_ and maximal voluntary ventilation (MVV), and the respiratory coefficient for CO_2_ calculated from V_E_ and carbon dioxide production (VE/VCO_2_) [7,8].

Therefore, the aim of the present study was to comprehensively evaluate long-term pulmonary function and exercise performance in children recovering from severe SARS-CoV-2-related respiratory disease and MIS-C approximately two years after hospitalization. In addition to conventional pulmonary function testing and CPET, we assessed diffusion capacity, ventilation homogeneity, and explored the relationship between CPET-derived indices (including OUES and BRI) and aerobic capacity.

## 2. Materials and Methods

**Study Population**

This was a single-center, prospective observational study, conducted between August 2023 and May 2024. Patients were consecutively identified from the institutional database of children previously hospitalized due to SARS-CoV-2-related respiratory disease or MIS-C and invited to participate in the follow-up assessment. A total of 48 participants older than 8 years who were capable of performing CPET were screened for this study. All participants were previously hospitalized at Institute for Mother and Child Healthcare of Serbia, and fully recovered from SARS-CoV-2 related respiratory disease (RD) or MIS-C. MIS-C was defined according to the Center for Disease Control and Prevention criteria as a febrile illness requiring hospitalization, with laboratory evidence of systemic inflammation, new onset of multisystem organ involvement, and evidence of recent SARS-CoV-2 infection or exposure, in the absence of an alternative diagnosis [9].

Inclusion criteria were:

- Children and adolescents aged ≥8 years.
- Previous hospitalization due to SARS-CoV-2-related respiratory disease (RD) or MIS-C.
- Complete clinical recovery from the acute illness before follow-up assessment.
- Ability to perform technically acceptable pulmonary function testing and maximal CPET.
- Written informed consent obtained from parents/legal guardians and assent from participants when appropriate.

Exclusion criteria were:

- Age < 8 years or unable to perform reliable pulmonary function testing or maximal CPET because of poor cooperation, anthropometric limitations, orthopedic or neurological conditions, or other contraindications.
- Patients who were not hospitalized during the acute SARS-CoV-2 infection or MIS-C episode.
- Persistent clinically significant cardiac abnormalities at follow-up (for participants with previous MIS-C).
- Failure to complete the study protocol or withdrawal of consent.

Of the 48 enrolled participants, 42 completed the study per protocol. The study protocol was approved by the Local Ethics Committee (decision number 8/8, 25 January 2023). Patients and their parents signed informed consents for the investigation.

**Study Procedures**

At the follow-up visit, all participants underwent a standardized evaluation including assessment of persistent symptoms, anthropometric measurements, pulmonary function testing, and cardiopulmonary exercise testing (CPET), performed during a single study visit. Persistent symptoms were assessed during a structured clinical interview with participants and their parents, focusing on fatigue, dyspnea, chest pain, palpitations, headache, muscle weakness, sleep disturbances, and anxiety/depressive symptoms. Measurements of lung function included spirometry, body plethysmography, diffusing capacity for carbon monoxide (DLCO), and multiple-breath washout (MBW). The reference equations used for spirometry were those of the Global Lung Initiative (GLI) [10].

Study participants were advised to follow their previous daily routine, including physical activity in days preceding CPET. Level of physical activity was estimated using standardized self-report questionnaires adapted for children and adolescents. Participants aged 8–14 years completed the Physical Activity Questionnaire for Children (PAQ-C), while those older than 14 years completed the Physical Activity Questionnaire for Adolescents (PAQ-A). Both validated questionnaires estimate habitual physical activity during the previous seven days. Responses were scored according to published guidelines, and a mean score ranging from 1 (low activity) to 5 (high activity) was calculated [11].

**Cardiopulmonary Exercise Test**

All participants were clinically stable, without any comorbidity that may have influenced CPET, including significant post-COVID heart or lung disease at the time of testing. Study participants who had no prior experience with CPET were familiarized with the equipment and procedures during routine clinical appointments prior to the commencement of the study. CPET was performed as a progressive exercise test on a cycle ergometer (Ergocard CPX Clinical, MGC Diagnostics, St. Paul, MN, USA), via modified Godfrey protocol until maximum effort was achieved. Participants breathed through a tightly sealed mask with electronically compensated dead space, connected to a flow sensor. Breath-to-breath analysis of ventilation, VO_2_, VCO_2_, and respiratory exchange ratio (RER) was continuously recorded during the test, which was designed to be completed within 8–10 min. Maximal voluntary ventilation (MVV) was calculated for each patient by multiplying the FEV_1_ value by 35.

Ventilation was expressed as V_E_/VCO_2_, V_E_/VCO_2_ slope, BRI (V_E_/MVV) and OUES (VO_2_/logV_E_) on anaerobic threshold (AT) and peak effort (W_peak_). AT was determined by the V-slope method. SaO_2_ and 12-lead ECG were recorded continuously, and blood pressure was measured before the test and every 2 min during the test. Stroke volume dynamics during exercise are represented by oxygen pulse (O_2_/HR). Normative values for OUES used for the purpose of this research were derived from Bongers et al. [12]. For other variables included in the analysis, normative values of Burnstein et al. were used [13]. Participants were encouraged throughout the test to achieve maximal effort. Peak exercise was considered maximal when clinical and physiological criteria were fulfilled, including participant exhaustion and an appropriate respiratory exchange ratio.

**Outcome Measures**

The primary outcome was exercise capacity assessed by peak oxygen uptake (VO_2_peak). Secondary outcomes included pulmonary function parameters, ventilatory efficiency indices (OUES and BRI), and the prevalence of persistent post-COVID symptoms.

**Statistical Analyses**

Data were analyzed using JASP statistical software (JASP Team (2026), version 0.96.0, University of Amsterdam, Amsterdam, The Netherlands). Statistical analyses were performed to evaluate the characteristics of the entire cohort and to compare the two study groups: the RD group and the MIS-C group. Continuous variables were summarized as mean ± standard deviation (SD) for normally distributed data or median with interquartile range (IQR) for non-normally distributed data, while categorical variables were presented as counts and percentages. Between-group comparisons of demographic characteristics, lung function parameters, and CPET data were performed using the independent samples t-test for normally distributed variables and the Mann–Whitney U test for non-normally distributed variables. Categorical variables were compared using the χ^2^ test or Fisher’s exact test when appropriate. To explore association between selected variables and CPET outcomes, univariable linear regression analyses were first performed. Variables that showed significant associations in univariable analysis were subsequently included in multivariable linear regression models to identify independent predictors. A *p*-value < 0.05 was considered statistically significant.

## 3. Results

This analysis included data from 42 children (25 males and 17 females). Participants were categorized into two groups: the group that presented with SARS-CoV-2 respiratory disease and the group with MIS-C. The mean participant age at the time of evaluation was 14.3 ± 3.1 years (range: 8.9–19.9 years). Children in the MIS-C group were significantly younger than those in the RD group, both at the time of hospitalization and at the time of study assessment. No significant differences between groups were observed in gender distribution, BMI, or length of hospitalization. The mean time between hospital discharge and study assessment was 22.8 ± 9.8 months, with a significantly shorter interval in the RD group (18.5 ± 7.1 months) in comparison to the MIS-C group (20.1 ± 9.9 months, *p* < 0.01). The self-reported level of previous physical activity was low in general and comparable between study groups.

All participants in the RD group required respiratory support, mostly low-flow oxygen therapy, while none required invasive mechanical ventilation.

In the MIS-C group, 11 participants (64.7%) had cardiac involvement. Reduced left ventricular ejection fraction (LVEF) was observed in 47.1% of participants, with cardiac inotropes required in 29.4%. No Kawasaki-like coronary involvement was detected. Immunomodulatory therapy, predominantly corticosteroids, was administered in 94% of participants with MIS-C.

Long COVID symptoms were present in 38.1% participants in whole cohort, significantly more often in the RD group compared with the MIS-C group (56.0% vs. 11.8%; *p* < 0.01). Fatigue was the most frequent symptom overall (28.6%) and was more common in the RD group (44.0% vs. 5.9%; *p* = 0.02). Other symptoms, including dyspnea, palpitations, chest pain, headache, muscle weakness, sleep difficulties, and anxiety/depression, were infrequent and did not differ significantly between groups.

Demographic and clinical data are presented in Table 1.

**Lung Function Tests Results**

In the whole cohort, the mean forced expiratory volume in the first second (FEV_1_) and forced vital capacity (FVC) were 95.5% and 90.3% predicted, respectively, with no statistically significant difference between the groups. Although all participants exhibited mild static hyperinflation, reflected by an elevated residual volume to total lung capacity ratio (RV/TLC median 139%), significant difference was not observed between the groups. DLCO and lung clearance index (LCI) values were within normal range for age and sex—Table 2.

**CPET Results**

Study participants in both study groups did not exhibit significant exercise intolerance. While the absolute value of W_peak_ was greater in the RD group (192.4 ± 61.5) compared to the MIS-C group (142.4 ± 43.8) (*p* < 0.01), this difference was not significant when compared to normative values for age and sex. There was no difference in VO_2_peak between groups. The AT was measured at a comparable level of VO_2_peak showing no significant deconditioning. Participants in the MIS-C group had a higher heart rate (HR) at W_peak_ (187.0 ± 7.6/min, 91.8 ± 6.6% predicted) in comparison to the RD group (180.0 ± 12.2/min, 96.0 ± 3.9% predicted) (*p* = 0.03 for HR at W_peak_, *p* = 0.01 for HR at W_peak_ % predicted). There was no significant difference in O_2_/HR between groups or significant O_2_/HR flattening. Low chronotropic index, which can suggest chronotropic incompetence, was present in 24% of subjects in the RD group, whereas none of the subjects in the MIS-C group exhibited this finding. However, the difference in distribution between the groups did not reach statistical significance (*p* = 0.07).

No ventilatory limitation was observed in the overall cohort, as indicated by V_E_/VCO_2_, V_E_/VCO_2_ slope, BRI, and OUES. In addition, no statistically significant differences were found between the study groups for these parameters. Univariable linear regression showed that OUES/kg, OUES/m2 and BRI as markers of ventilatory limitation correlate significantly with peak exercise capacity in both RD and MIS-C groups. Multivariable regression analysis demonstrated that BRI and OUES/kg are strong positive predictors of aerobic capacity among parameters that describe ventilatory limitation in both the MIS-C and RD groups. In the MIS-C group, the model explained 80.2% of the variance (adjusted R^2^ = 0.802, F(2,13) = 31.44, *p* < 0.001), and in the RD group the model explained 81.3% of the variance (adjusted R^2^ = 0.813, F(2,22) = 53.23, *p* < 0.001)—Table 3.

Although the time from infection/MIS-C to CPET differed significantly between groups, stratified analyses performed separately within the COVID and MIS-C groups, as well as between them, using a 24-month cut-off, showed no significant differences in CPET or lung function parameters—Table 4.

## 4. Discussion

The present study provides a comprehensive long-term assessment of respiratory function and cardiopulmonary exercise performance in children recovering from severe SARS-CoV-2-related respiratory disease and MIS-C. Several important findings emerged. First, objective measures of pulmonary function, gas exchange, ventilation homogeneity, and cardiopulmonary exercise performance remained within the normal range nearly two years after hospitalization, with no evidence of ventilatory limitation or impaired aerobic capacity, indicating favorable long-term physiological recovery. Second, persistent subjective symptoms, particularly fatigue, continued to be reported by a substantial proportion of participants despite preserved functional capacity, suggesting that recovery of physiological function may precede complete symptom resolution. Finally, the study identified CPET-derived indices, including OUES normalized to body weight and body surface area and the breathing reserve index, as strong predictors of aerobic capacity, highlighting their potential value in the functional assessment of children recovering from severe SARS-CoV-2-related illness.

One of the most important findings of the present study is the apparent dissociation between objective functional recovery and the persistence of subjective symptoms. Although a substantial proportion of children, particularly those recovering from SARS-CoV-2-related respiratory disease, continued to report fatigue and other persistent symptoms at long-term follow-up, pulmonary function and cardiopulmonary exercise performance remained within the normal range. These findings suggest that persistent symptoms in pediatric post-COVID patients do not necessarily reflect ongoing impairment of respiratory mechanics or aerobic exercise capacity. Similar observations have been reported in adults, in whom preserved CPET parameters despite exertional symptoms have been attributed to mechanisms other than primary cardiopulmonary dysfunction, including physical deconditioning or dysfunctional breathing, although the relative contribution of these mechanisms remains debated [4,14,15,16,17]. In contrast, a previous pediatric study evaluating children within the first year after MIS-C reported lower VO_2_peak and higher VE/VCO_2_ slope values, while another cohort demonstrated an association between persistent fatigue and impaired pulmonary function and exercise performance [5,18]. These findings were not confirmed in our cohort. One possible explanation is the substantially longer follow-up period in our study (approximately 22 months compared with 8–12 months in previous reports), suggesting that objective respiratory and exercise abnormalities may gradually resolve over time, even though subjective symptoms may persist in some patients.

An additional noteworthy finding of the present study is the comparable long-term functional restoration observed in children recovering from SARS-CoV-2-related respiratory disease and MIS-C, despite the distinct pathophysiological mechanisms characterizing these conditions during the acute phase. Although persistent symptoms, particularly fatigue, were reported more frequently by children with respiratory disease, integrative functional responses were remarkably similar between the two groups. This finding is particularly noteworthy given the markedly different patterns of acute organ involvement characterizing respiratory disease and MIS-C [19]. Such an observation suggests that the predominant pattern of acute organ involvement may not necessarily determine long-term respiratory or exercise performance in pediatric patients. Rather, both groups appear to follow a favorable trajectory of physiological recovery, with restoration of pulmonary function and aerobic capacity over time. Several explanations have been proposed, including autonomic dysfunction, persistent low-grade inflammation, impaired peripheral oxygen utilization, altered perception of exertion, and physical deconditioning, although the relative contribution of these mechanisms remains uncertain [19,20]. Since these factors were not directly evaluated in our study, no causal inferences can be drawn. Persistent fatigue is likely multifactorial and may also be influenced by psychological, behavioral, endocrine, lifestyle-related, or other non-cardiopulmonary factors that were beyond the scope of the present study. Nevertheless, the normal functional parameters, exercise capacity, and absence of clinically significant ventilatory limitation observed in our cohort argue against persistent cardiopulmonary impairment as the predominant explanation for ongoing symptom burden nearly two years after hospitalization.

Cardiopulmonary exercise testing provided additional insight into the mechanisms underlying exercise performance in our cohort. Previous studies in adults with Long COVID have reported chronotropic incompetence and reduced stroke volume as potential contributors to exercise intolerance [21]. In children recovering from MIS-C, mild O_2_ pulse flattening has also been described despite the absence of overt chronotropic incompetence [22]. In contrast, our patients demonstrated preserved aerobic capacity, normal oxygen pulse, and no evidence of chronotropic incompetence according to the chronotropic index. These results suggest preserved integrative cardiovascular adaptation to exercise despite subtle differences in heart rate response. Although heart rate at peak exercise was slightly higher and heart rate reserve correspondingly lower in the MIS-C group, these findings were not accompanied by reduced exercise performance and are therefore unlikely to represent clinically relevant cardiovascular limitation.

Beyond conventional CPET variables, our findings also highlight the potential clinical utility of OUES normalized to body weight and body surface area, as well as BRI, as complementary markers of aerobic capacity [23]. Unlike peak VO_2_, OUES reflects the efficiency of oxygen uptake in relation to ventilation throughout incremental exercise and is considered less dependent on maximal patient effort, making it a robust parameter for evaluating exercise performance, particularly in pediatric populations [23]. Previous studies have demonstrated a strong association between OUES and peak VO_2_ in healthy children, supporting its role as a reliable surrogate of aerobic fitness [23,24]. Its reduced dependence on maximal exercise effort may be particularly advantageous in pediatric populations, in whom achieving a truly maximal test is often challenging [23,24]. Our findings extend these observations to children recovering from severe SARS-CoV-2-related respiratory disease and MIS-C, in whom normalized OUES remained a strong predictor of aerobic capacity despite the absence of overt functional impairment. Similarly, the breathing reserve index showed a strong association with peak VO_2_, suggesting that CPET-derived indices may provide additional physiological information beyond conventional exercise parameters. Although these findings require confirmation in larger prospective cohorts, they support the incorporation of OUES and BRI into the comprehensive functional assessment of children following severe SARS-CoV-2-related illness.

Taken together, our findings suggest that comprehensive functional assessment provides a more reliable evaluation of long-term recovery than symptom reporting alone. This distinction may have important implications for clinical decision-making, helping to guide individualized follow-up strategies and avoid unnecessary cardiopulmonary investigations in patients with reassuring favorable functional parameters. Future studies integrating cardiopulmonary exercise testing with assessments of autonomic function, peripheral muscle performance, inflammatory biomarkers, and patient-reported outcomes may further clarify the mechanisms responsible for persistent symptom burden and improve the long-term management of pediatric post-COVID syndrome.

The present study has several limitations that should be considered when interpreting the findings. First, this was a single-center study with a relatively modest sample size, which limited statistical power for subgroup analyses and may have reduced the ability to detect smaller between-group differences. Second, only children who required hospitalization for SARS-CoV-2-related respiratory disease or MIS-C were included; therefore, our findings cannot be generalized to children with milder disease managed in the outpatient setting. Third, pre-infection pulmonary function and cardiopulmonary exercise testing data were unavailable, precluding direct within-subject comparisons and limiting conclusions regarding individual changes attributable to SARS-CoV-2 infection. In addition, although stratified analyses according to follow-up duration did not demonstrate significant differences in pulmonary function or CPET parameters, the longer interval between hospitalization and study assessment in the MIS-C group may still have influenced symptom reporting and the extent of functional recovery. Furthermore, persistent symptoms were assessed by structured self-report during clinical follow-up, introducing the possibility of recall and reporting bias.

Despite these limitations, the present study has several important strengths. It represents one of the few studies to provide a long-term respiratory assessment in children following severe SARS-CoV-2 infection and MIS-C by combining spirometry, body plethysmography, diffusing capacity, multiple-breath washout, and cardiopulmonary exercise testing within the same cohort. Moreover, the relatively long follow-up period, extending to nearly two years after hospitalization, enabled evaluation of both objective functional recovery and the persistence of subjective symptoms. Future multi-center longitudinal studies including larger patient cohorts, healthy age-matched controls, and serial functional assessments are warranted to better characterize the trajectory of respiratory recovery and persistent post-COVID symptoms in the pediatric population.

## 5. Conclusions

Children demonstrated preserved pulmonary function and exercise capacity nearly two years after severe SARS-CoV-2-related respiratory disease or MIS-C, with no evidence of clinically significant ventilatory limitation. Despite these favorable objective findings, persistent subjective symptoms remained present in a substantial subset of patients, particularly among those with respiratory disease, suggesting that symptom resolution may lag behind objective functional recovery. In addition, OUES normalized to body weight and BSA, together with the breathing reserve index, emerged as strong predictors of aerobic capacity, supporting their potential value as complementary markers in the functional assessment of children recovering from SARS-CoV-2-related illness.

## Figures and Tables

**Table 1:** Demographic and clinical data.

	Whole Cohort N = 42	RD N = 25	MIS-C N = 17	*p*-Value
**Male gender, n (%)**	25 (59.5)	15 (60)	10 (58.8)	0.94
**Female gender, n (%)**	17 (40.5)	10 (40.0)	7 (41.2)	
**Age at the time of** **infection/MIS-C, mean ± SD**	12.4 ± 3.2	13.9 ± 2.7	10.0 ± 2.3	**<0.01**
**Age at the time of testing, mean ± SD**	14.3 ± 3.1	15.5 ± 2.8	12.5 ± 2.5	**<0.01**
**Months passed since the** **infection/MIS-C to testing,** **mean ± SD**	22.8 ± 9.8	18.5 ± 7.1	29.1 ± 9.9	**<0.01**
**BMI, mean ± SD**	21.71 ± 5.28	22.54 ± 5.79	20.48 ± 4.31	0.22
**Level of physical activity,** **median (IQR)**	2.2 (1.4–2.7)	2.0 (1.3–2.6)	2.6 (2.2–2.8)	0.14
**Hospitalization length,** **median (IQR)**	9 (8–12)	7.0 (3–9.75)	10 (8–12)	0.11
**Cardiac involvement, n (%)**	11 (26.2)	0 (0)	11 (64.7)	**<0.01**
**Antibiotics, n (%)**	32 (76.2)	17 (68)	15 (88.2)	0.16
**Corticosteroids, n (%)**	22 (52.4)	6 (24)	16 (94.1)	**<0.01**
**Long COVID symptoms, n (%)**	16 (38.1)	14 (56.0)	2 (11.8)	**<0.01**
**Fatigue**	12 (28.6)	11 (44.0)	1 (5.9)	**0.02**
**Dyspnea**	2 (4.8)	2 (8.0)	0 (0)	0.52
**Palpitations**	4 (9.5)	3 (12.0)	1 (5.9)	1.00
**Chest pain**	2 (4.8)	1 (4.0)	1 (5.9)	1.00
**Headache**	4 (9.5)	4 (16.0)	0 (0)	0.28
**Muscle weakness**	1 (2.4)	1 (4.0)	0 (0)	1.00
**Sleep difficulties**	2 (4.8)	2 (8.0)	0 (0)	0.52
**Anxiety/Depression**	1 (2.4)	1 (4.8)	0 (0)	1.00

RD—respiratory disease; MIS-C—multisystem inflammatory syndrome; BMI—body mass index.

**Table 2:** Lung function results.

	Whole Cohort N = 42	RD N = 25	MIS-C N = 17	*p*-Value
**FEV_1_ %, median (IQR)**	95.5 (90.3–99.0)	97.0 (92.0–99.0)	94.0 (86.0–98.0)	0.37
**FVC %, median (IQR)**	90.0 (84.0–98.0)	90.0 (84.0–98.0)	89.0 (85.0–98.0)	0.94
**RV/TLC %, median (IQR)**	139.0 (118.0–183.0)	141.0 (121.0–170.0)	137.0 (104.0–219.0)	0.89
**DLCO %, median (IQR)**	97.0 (88.0–108.0)	95.5 (84.8–111.8)	97.0 (90.0–105.0)	0.57
**KCO %, median (IQR)**	105.0 (97.0–112.0)	104.5 (97.8–112.8)	105.0 (97.0–112.0)	1.00
**LCI %, median (IQR)**	5.5 (4.8–6.8)	5.5 (4.7–6.3)	5.9 (4.8–6.9)	0.94

**Table 3:** Multivariable linear regression analysis of predictors of VO_2_peak (mL/kg/min) in RD and MIS-C groups.

Variable	RD β (95% CI)	*p*-Value	MIS-C β (95% CI)	*p*-Value
**Intercept**	−3.73 (−10.82–3.37)	0.29	−4.49 (−14.72–5.73)	0.36
**BRI**	21.94 (10.31–33.58)	<0.001	12.88 (1.11–24.66)	0.03
**OUES/kg**	703.29 (478.85–927.73)	<0.001	942.40 (614.34–1270.45)	<0.001

BRI—breathing reserve index; OUES/kg—oxygen uptake efficiency slope normalized to body mass.

**Table 4:** CPET results.

CPET Parameter, Mean ± SD	RD N = 25	MIS-C N = 17	*p*-Value
**W_peak_ (watt)**	192.4 ± 61.5	142.4 ± 43.8	**<0.01**
**W_peak_ % predicted**	99.0 ± 19.1	89.3 ± 15.4	0.87
**VO_2_peak**	1892.0 ± 600.9	1608.0 ± 443.6	0.10
**VO_2_peak/kg**	30.44 ± 9.14	32.47 ± 7.95	0.46
**VO_2AT_/kg % predicted**	63.4 ± 24.4	68.7 ± 24.2	0.49
**VO_2AT_/VO_2_peak**	54.9 ± 15.7	54.3 ± 12.5	0.89
**Vt_peak_**	1.74 ± 0.53	1.37 ± 0.57	**0.04**
**Vt_peak_ %predicted**	93.0 ± 19.7	89.1 ± 23.9	0.56
**Ve_peak_**	65.6 ± 23.5	55.9 ± 16.6	0.15
**Ve_peak_ % predicted**	65.8 ± 18.2	66.06 ± 16.6	0.96
**OUES/kg**	0.031 ± 0.008	0.031 ± 0.008	0.78
**OUES/m^2^**	1.107 ± 0.220	1.054 ± 0.198	0.43
**Breathing reserve**	50.7 ± 14.4	45.8 ± 15.5	0.29
**Breathing reserve % predicted**	163.8 ± 42.3	166.8 ± 53.0	0.84
**BRI_peak_**	0.56 ± 0.16	0.62 ± 0.18	0.35
**BRI_AT_**	0.21 ± 0.09	0.22 ± 0.10	0.74
**RER**	1.29 ± 0.16	1.25 ± 0.19	0.39
**HR_peak_**	180.0 ± 12.2	187.0 ± 7.6	**0.03**
**HR_peak_ % predicted**	91.8 ± 6.6	96.0 ± 3.9	**0.01**
**VO_2_/HR_peak_**	10.6 ± 3.4	8.7 ± 2.5	0.07
**VO_2_/HR_peak_ % predicted**	103.1 ± 31.3	96.8 ± 23.7	0.49
**V_E_/VCO_2AT_**	26.16 ± 4.09	25.38 ± 2.65	0.50
**V_E_/VCO_2AT_ % predicted**	85.2 ± 12.6	79.4 ± 7.4	0.07
**V_E_/VO_2AT_**	23.7 ± 4.2	24.2 ± 2.8	0.66
**V_E_/VO_2AT_ % predicted**	78.1 ± 12.9	77.6 ± 9.2	0.89
**V_E_/VCO_2_ slope**	25.1 ± 3.5	27.2 ± 5.1	0.13
**V_E_/VCO_2_ slope % predicted**	88.4 ± 11.0	90.3 ± 15.9	0.66
**VLI**	34.3 ± 19.0	27.5 ± 20.8	0.28

## Data Availability

The original contributions presented in this study are included in the article. Further inquiries can be directed to the corresponding author.

## Abbreviations

The following abbreviations are used in this manuscript:

SARS-CoV-2	Severe Acute Respiratory Syndrome Coronavirus 2
MIS-C	Multisystem Inflammatory Syndrome in Children
PFTs	Pulmonary Function Tests
CPET	Cardiopulmonary Exercise Testing
VO_2_	Oxygen Consumption
VE	Minute Ventilation
OUES	Oxygen Uptake Efficiency Slope
BSA	Body Surface Area
BM	Body Mass
BRI	Breathing Reserve Index
MVV	Maximal Voluntary Ventilation
VCO_2_	Carbon Dioxide Output
VE/VCO_2_	Ventilatory Equivalent for Carbon Dioxide
AT	Anaerobic Threshold
Wpeak	Peak Work Rate
SaO_2_	Arterial Oxygen Saturation
ECG	Electrocardiogram
O_2_/HR	Oxygen Pulse
GLI	Global Lung Initiative
PAQ-C	Physical Activity Questionnaire for Children
PAQ-A	Physical Activity Questionnaire for Adolescents
FEV_1_	Forced Expiratory Volume in 1 Second
FVC	Forced Vital Capacity
DLCO	Diffusing Capacity for Carbon Monoxide
MBW	Multiple-Breath Washout
LCI	Lung Clearance Index
RV/TLC	Residual Volume to Total Lung Capacity Ratio
KCO	Transfer Coefficient of the Lung for Carbon Monoxide
BMI	Body Mass Index
RD	Respiratory Disease
LVEF	Left Ventricular Ejection Fraction
HR	Heart Rate
RER	Respiratory Exchange Ratio
Vt	Tidal Volume
VLI	Ventilatory Limitation Index
